# Auxiliary Subunits: Shepherding AMPA Receptors to the Plasma Membrane

**DOI:** 10.3390/membranes4030469

**Published:** 2014-08-08

**Authors:** Simon C. Haering, Daniel Tapken, Steffen Pahl, Michael Hollmann

**Affiliations:** 1 Department of Biochemistry I—Receptor Biochemistry, Ruhr University Bochum, Universitätsstraße 150, 44780 Bochum, Germany; E-Mails: simon.koesters@rub.de (S.C.H.); daniel.tapken@rub.de (D.T.); steffen.pahl@rub.de (S.P.); 2 Graduate School of Chemistry and Biochemistry, Ruhr University Bochum, Universitätsstraße 150, 44780 Bochum, Germany; 3 Ruhr University Bochum Research School Plus, Ruhr University Bochum, Universitätsstraße 150, 44780 Bochum, Germany; 4 International Graduate School of Neuroscience, Ruhr University Bochum, Universitätsstrraße 150, 44780 Bochum, Germany

**Keywords:** AMPA, glutamate receptor, trafficking, auxiliary subunit, TARP, cornichon homologue, CNIH, shisa, SynDIG, CKAMP, GSG1L

## Abstract

Ionotropic glutamate receptors (iGluRs) are tetrameric ligand-gated cation channels that mediate excitatory signal transmission in the central nervous system (CNS) of vertebrates. The members of the iGluR subfamily of α-amino-3-hydroxy-5-methyl-4-isoxazole propionic acid (AMPA) receptors (AMPARs) mediate most of the fast excitatory signal transmission, and their abundance in the postsynaptic membrane is a major determinant of the strength of excitatory synapses. Therefore, regulation of AMPAR trafficking to the postsynaptic membrane is an important constituent of mechanisms involved in learning and memory formation, such as long-term potentiation (LTP) and long-term depression (LTD). Auxiliary subunits play a critical role in the facilitation and regulation of AMPAR trafficking and function. The currently identified auxiliary subunits of AMPARs are transmembrane AMPA receptor regulatory proteins (TARPs), suppressor of lurcher (SOL), cornichon homologues (CNIHs), synapse differentiation-induced gene I (SynDIG I), cysteine-knot AMPAR modulating proteins 44 (CKAMP44), and germ cell-specific gene 1-like (GSG1L) protein. In this review we summarize our current knowledge of the modulatory influence exerted by these important but still underappreciated proteins.

## 1. Introduction

The family of iGluRs consists of 18 different subunits found in mammals which are categorized into four subfamilies based on pharmacological properties, biological function, and sequence homology. The four subunits GluA1 through GluA4 constitute the subfamily of AMPARs which assemble as homo- or heterotetramers to form functional receptors. 

Synaptic plasticity is the basis of learning and memory formation. LTP and LTD—the two major mechanisms of synaptic plasticity at excitatory synapses—involve the regulation of the number of AMPA receptors in the active zone of synapses. LTP, the process of synapse strengthening, is accompanied by an increase in the number of active AMPARs at the PSD. The current knowledge about the general mechanism of LTP generation can be summarized in a three-step model: (i) exocytosis of AMPARs at the dendritic shaft or the spine lateral to the PSD; (ii) lateral diffusion of AMPARs to the PSD; and (iii) anchoring of the AMPARs at the PSD. LTD, the opposite process, leads to the endocytosis of AMPARs and thus to a reduced number of AMPARs at the PSD [1]. 

On their way from the ER to the PSD, AMPARs interact temporarily, either directly or indirectly, with various proteins, including chaperones, stabilizers, mediators of vesicular delivery, and anchoring proteins. These interacting proteins, which include for example GRIP1, KIF1A, KIF5, Rab8, Liprin-α, NEEP21, GRASP-1, Rab4, and Stx13, and their functions in trafficking pathways of AMPARs have recently been reviewed by Anggono and Huganir and are not described here [2]. Instead, we focus on auxiliary subunits of AMPARs: highly specialized, non-transient binding partners that—besides their role in receptor trafficking—also modulate pharmacological and electrophysiological properties of AMPARs.

### 1.1. The Discovery of TARPs and Their Functional Properties

**Figure 1:**
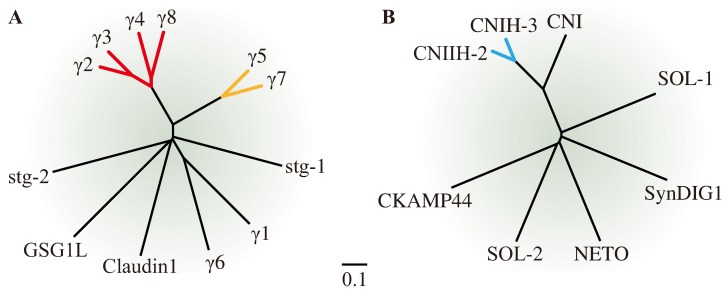
(**A**) Phylogenetic tree of all tetraspanin-resembling four transmembrane domain-containing auxiliary subunits: type I TARPs (red), type II TARPs (orange), stg-1, stg-2, γ1, γ6, GSG1L, and claudin1 as a representative of the most closely related group of non-γ subunit proteins. (**B**) Phylogenetic tree of all non-tetraspanin-resembling auxiliary subunits with less than four transmembrane domains: CNIHs (blue), CNI, NETO, SOL-1, SynDIGI, SOL-2, CKAMP44. The ClustalW protein alignment was used for calculation and the Phylodendron program for the tree generation. Scale indicates number of exchanges per position along the branches connecting two proteins.

The first AMPAR auxiliary subunit—stargazin—was discovered in the epileptic and ataxic stargazer mouse mutant, which lacks functional AMPARs in cerebellar granule cell synapses. In stargazer mice, the expression of the stargazin gene is disrupted by a spontaneous mutation in both its alleles. Stargazin is a four-transmembrane-domain protein showing structural and sequence similarity to the γ1 subunit of voltage-activated calcium channels, and was therefore alternatively named γ2 [3]. Eventually, several additional members of the γ subunit family, sharing sequence similarity with γ1 and γ2, were shown to also share functional similarity with γ2. These proteins defined the new family of TARPs, which was later subdivided on the basis of functional differences and sequence homologies (see below) into the type I TARPs comprising the subunits γ2, γ3, γ4, and γ8, and the type II TARPs, γ5 and γ7 (see Figure 1/Table 1). The related proteins γ1 and γ6 do not belong to the TARP family because they do not modulate AMPARs (see Table 1). TARPs are non-pore-forming integral membrane proteins with four transmembrane domains (thus resembling tetraspanins) that directly interact with AMPARs [4,5,6,7,8,9,10]. They are expressed differentially all over the brain, and γ2 in particular is expressed in every type of neuron [11]. Moreover, almost all tissues and cell types in the brain express more than one type I TARP subunit, with the exception of cerebellar granule cells, which express only one type I TARP, γ2 [11,12,13], plus one type II TARP, γ7 [14]. TARPs alter the maturation and trafficking of AMPARs; however, they do not act as classical chaperones because they are more than just temporary binding partners [10,13,15,16]. They remain bound to the receptor within the postsynaptic membrane and modulate the receptor's pharmacological and electrophysiological properties, including agonist efficacy, activation time, deactivation rate, and desensitization rate [17,18,19,20,21,22]. In doing so, they are exquisitely specific for AMPARs, not affecting other iGluRs such as kainate or NMDA receptors [23].

The TARP γ2 is present in many different species, vertebrates as well as invertebrates. In the invertebrate *Caenorhabditis elegans* (*C. elegans*), the stargazin homologs stg-1 and stg-2 facilitate the currents of a glutamate receptor homolog that is assembled from GLR-1 subunits. The SOL-1 and SOL-2 proteins represent another group of auxiliary subunits present in *C. elegans.* GLR-1 has to be coexpressed with both, stg-1 and SOL-1, to achieve detectable glutamate-activated currents in heterologous expression systems [24,25,26,27]. SOL-1 and stg-1 bind directly to the GLR-1 receptor [25,28] but, in contrast to the vertebrate TARPs, they do not influence its plasma membrane expression (see Table 2) [26,27].

### 1.2. More Recently Discovered AMPAR Auxiliary Subunits 

**Figure 2:**
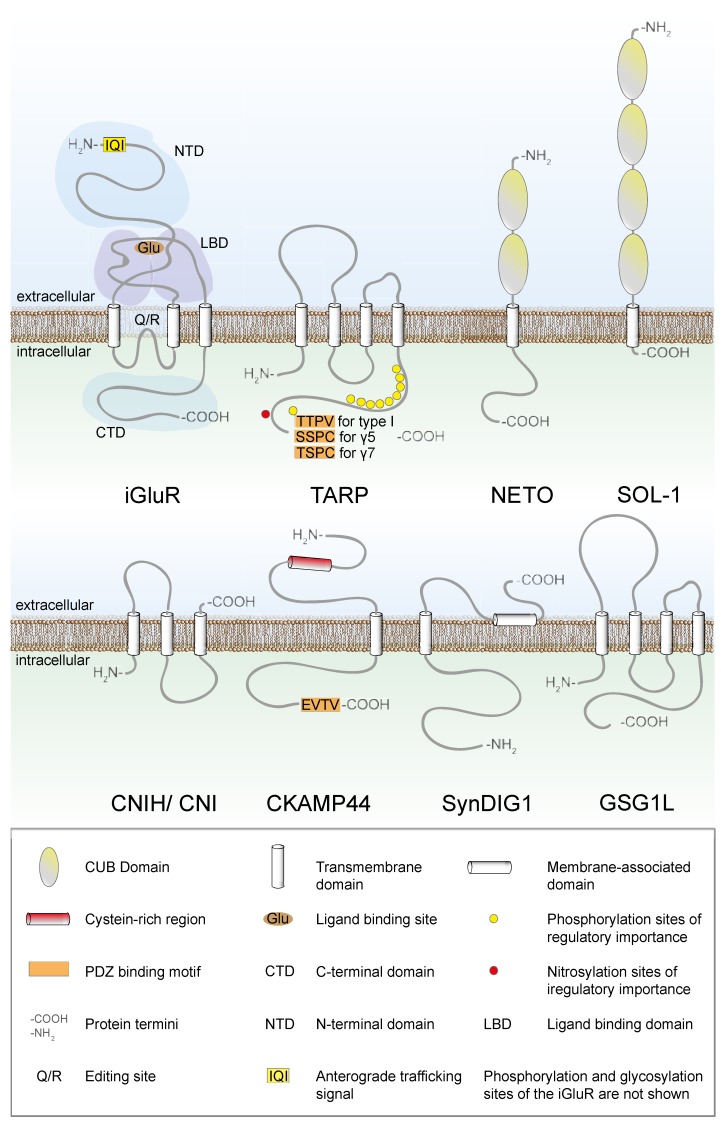
Schematic structures of iGluRs, TARPs, NETO, SOL-1, CNIH/CNI, CKAMP44, SynDIG1, and GSG1L.

Proteomic analyses led to the discovery of CNIHs as another class of AMPAR auxiliary subunits (see Table 2) [29]. CNIHs are small, three-transmembrane-domain proteins that were first discovered as cornichon protein (CNI) in *Drosophila* as participants in the Golgi secretory pathway (see Figure 2). When CNIHs are overexpressed together with AMPA receptors, they also localize in the Golgi apparatus [30,31]. CNIHs, like TARPs, directly bind to AMPARs. Reportedly, each of the AMPAR subunits GluA2, GluA3, and GluA4 has only a single site that can bind either a CNIH or a TARP, resulting in competition between CNIH and TARP binding [29,32]. Only GluA1 was suggested to have two different binding sites for CNIHs and TARPs [32]. Consequently, it was initially believed that there is a pool of AMPARs solely associated with CNIHs and another pool associated with TARPs [29,33]. There is even evidence that a majority (70%) of all AMPARs may be associated with CNIHs and not with the prototypic auxiliary subunits, the TARPs [29]. However, other investigations revealed that CNIHs predominantly alter electrophysiological properties and trafficking of GluA1 rather than of the other AMPARs [32]. The observed preferred interaction between CNIH-2 and a GluA1/γ8 complex supports these findings [34]. Other, more recent studies, suggest a different, semi-competitive model with two pairs of distinct binding sites for TARPs and CNIHs per AMPAR tetramer. At one of these pairs, γ2 and γ3 compete for binding with CNIHs, whereas the other pair can bind all type I TARPs but not CNIHs [6]. CNIH-2/3 influence the electrophysiological and pharmacological properties of AMPARs; however, they do this to a greater extent in heterologous systems (up to 10-fold increase in current amplitude) than *in vivo* (no significant modulation of current amplitude) [31,35]. The modulation of AMPAR trafficking by CNIHs appears to occur independently of that by TARPs [29,31,34]. Thus, it is conceivable that CNIHs and TARPs promote two different trafficking pathways for AMPARs [36,37].

SynDIG1 was identified in a microarray analysis as an additional AMPAR auxiliary subunit (see Table 2) [38]. It directly interacts with and modulates AMPARs, and it additionally has an impact on NMDARs. There are several reports stating that AMPAR activity is regulated via SynDIG1, but the effect of SynDIG1 on the trafficking of AMPARs is not well understood [39,40,41]. On the one hand, the knockout of SynDIG1 down-regulates the AMPAR content in developing synapses by 50%, and SynDIG1 overexpression increases the amplitude of mEPSPs as well as their frequency up to a factor of 1.5–2 in hippocampal cells [40]. On the other hand, there is evidence from acute hippocampal brain slices that SynDIG1 does not have any influence on the surface expression level of AMPARs at all [41]. Thus, the question whether SynDIG1 should be called an auxiliary GluR subunit remains to be answered conclusively.

CKAMP44 and CKAMP52 are members of the shisa protein family, a family of signal transduction pathway-modulating proteins. CKAMP44 and CKAMP52 are auxiliary subunits that can coimmunoprecipiate with AMPARs, proving a direct AMPAR-CKAMP44 interaction (see Table 2) [42]. CKAMP44 influences the electrophysiological properties of AMPARs: Its coexpression with AMPARs in the *Xenopus laevis* heterologous expression system leads to a nearly complete loss of AMPAR currents and a two-fold increased deactivation rate of the remaining current [42,43,44]. Overexpression of CKAMP44 does not influence the plasma membrane expression of AMPARs *in vivo* or in heterologous systems [42,43].

A recently reported novel protein that influences AMPARs is GSG1L protein, a relative of the claudin tight junction proteins. It boosts the surface expression of AMPARs in heterologous systems and directly interacts with AMPARs and their complexes (see Table 2). GSG1L additionally influences the electrophysiological properties of the receptor, such as desensitization and activation [6,45]. 

Another family of iGluR auxiliary subunits are the NETO proteins, which were first thought to influence the NMDARs [46]. Meanwhile it has become clear that they actually modulate kainate receptor subtypes of iGluRs in vertebrates [47]. However, the *Drosphila* NETO protein seems to be important for the clustering of iGluRs at the neuromuscular junctions. It is therefore a good example of the versatile modulation of iGluRs by auxiliary subunits through vertebrate and invertebrate animals [48].

## 2. Trafficking of AMPARs between the ER and the Golgi Apparatus

### 2.1. TARPs and AMPAR Trafficking between the ER and the Golgi Apparatus

The four AMPAR subunits GluA1 through GluA4 have to assemble into hetero- or homotetramers to form functional receptors, a process that takes place in the ER. First, two monomers form a dimer, and then two dimers assemble to form the tetramer [49]. The TARP–receptor interaction starts within the ER and requires the tetrameric structure of the receptor [8,13]. In the ER, TARPs act as chaperones to prevent the ER export of incorrectly folded receptors [13,50]. However, whereas classical chaperones are only transient binding partners within the ER, TARPs remain bound to the receptor on its way through the Golgi apparatus to the plasma membrane and finally the active zone [15]. In this way, type I TARPs are crucial for the ER export of the receptors, which consequently become enriched in the ER when type I TARPs are lacking [30,50,51,52]. The overexpression of AMPARs alone leads to their accumulation in the cell body, and it is only the coexpression with γ2 that decreases this somatic accumulation of AMPARs [53]. However, the TARPs do not influence AMPAR maturation and trafficking indiscriminately but rather in a receptor subunit-specific way [13,50,54]. For example, in Golgi cells γ2 and γ3 can alter the composition of the AMPARs that reach the surface by promoting the surface expression exclusively of GluA2-containing AMPARs [54]. There are two possibilities of how TARPs promote the ER export of AMPARs. The first possibility is that they mask an ER retention signal of the receptor, the second is that they have their own ER export signals that trigger transport of the entire AMPAR-TARP complex [13,50,55]. Recent findings demonstrating the importance of the TARPs’ C-terminus for ER export support the second possibility, or at least a combination of the two mechanisms [56]. The AMPAR GluA1 itself has an anterograde trafficking signal (IQI) at position 7–9 in the amino acid sequence (see Figure 2), *i.e.*, at the far amino terminal end of the protein [57]. More recent findings like the importance of the TAPR`s C-terminus for the trafficking suggest, however, that this signal may not be sufficient for successful plasma membrane delivery [56]. In addition to their function in folding and assembly, type I TARPs are suspected to influence the glycosylation state of the receptor. The AMPARs of stargazer mice show an immature ER-type glycosylation pattern, which indicates that type I TARPs are important for the correct glycosylation of AMPARs [13,50]. 

### 2.2. Other Auxiliary Subunits Impacting Trafficking between the ER and the Golgi Apparatus

The cornichon proteins are well known for their localization in the ER and their function as ER exporter proteins in *Drosophila* [30,58,59,60]. The CNIHs act as ER exporters just like their *Drosophila* homologues and are highly enriched in the ER when overexpressed [31]. CNIH-2 is involved in the COPII-mediated vesicular transport of proteins from the ER to the Golgi apparatus and cycles between these two organelles [61]. However, when CNIH-2 is accompanied by AMPARs, it forms a functional complex with them that exits this cycle to travel to the plasma membrane [61]. CNIHs enhance AMPAR export from both the ER and the Golgi apparatus [58,61]. However, the functional consequences of CNIH assembly with AMPARs in the ER, like those of TARP assembly, are more varied. CNIHs modulate protein maturation in the ER by altering the glycosylation pattern of the receptors, as observed, for example, for GluA2 and CNIH-2 [61]. Additionally, CNIH-2 competitively reduces the TARP stoichiometry in AMPAR-TARP complexes and assembles preferentially with AMPAR-TARP complexes that include γ8 [62].

AMPARs interact with a large number of proteins in the ER and Golgi apparatus. However, just a few of these AMPAR-interacting proteins interact specifically with the AMPAR—auxiliary subunit complex [2,63,64]. AP-4 is involved in the vesicular transport of AMPAR-TARP complexes, and its knockout leads to mislocalization of the complexes and their accumulation in autophagosomes. AP-4 is additionally involved in the polarized transport of AMPAR-TARP complexes to the somatodendritic domain of Purkinje cells [64]. LC2 binds to fully glycosylated GluA2 subunits in complex with γ2 on their way from the Golgi apparatus to the plasma membrane. It associates with MAP1A and is responsible for neuronal differentiation and microtubule dynamics [63]. Unlike TARPs, however, AP-4 and LC2 are not auxiliary subunits in the sense of the definition given in the introduction because they are merely transient binding partners of the AMPAR complex and do not alter receptor properties.

## 3. Trafficking of AMPARs to the Plasma Membrane

### 3.1. The Role of TARPs in Trafficking to the Plasma Membrane

Regulation of the plasma membrane expression of AMPARs is perhaps the most obvious, although not the only, characteristic property of most AMPAR auxiliary subunits [3]. As mentioned above, TARPs, CNIHs, SynDIG, and GSG1L all alter the surface expression of AMPARs. TARPs in particular are crucial for the plasma membrane delivery of AMPARs in the CNS [19,20,65,66]. Overexpression of γ2 enhances AMPAR surface expression *in vivo* [13,66] and in heterologous systems like *Xenopus laevis* oocytes by a factor of ten [23,67].

In stargazer mice, which lack the prototypical TARP γ2, the plasma membrane delivery of AMPARs in granule cells is disturbed but can be rescued by the expression of any other type I TARP [13]. In fact, the ability to restore AMPAR function in stargazer cerebellar granule cells was the criterion used to define the type I TARP subfamily. Thus, type I TARPs seem to have at least partly redundant functions. This can also be observed in several other cell types of TARP knockout mice. For example, Golgi cells express γ2 and γ3, and, consequently, a single knockout of either of these subunits does not influence the surface expression of AMPARs. It takes a double knockout of γ2 and γ3 to produce a deficit in AMPAR plasma membrane delivery in these cells [54]. This functional redundancy is the main reason why the stargazer mutant is the only single TARP knockout strain that shows a phenotype: All CNS cell types express at least two different, redundant TARPs, with the exception of cerebellar granule cells, which express only γ2 [54] out of the type I TARP subfamily. Granule cells also express the type II TARP γ7; however, this subunit cannot efficiently compensate for the lack of γ2. In addition to the basic AMPAR delivery to the surface, type I TARPs are also required for the fast cycling of AMPARs into and out of the plasma membrane, and thus underlie the mechanisms of LTP and LTD [50].

Type II TARPs differ from type I TARPs in sequence and function, but the functional differences are not as pronounced as initially thought. The type II TARP γ5 does not alter surface trafficking of AMPARs [7,13,68], its influence appears to be limited to a modulation of their electrophysiological properties [69]. However, the second type II TARP, γ7, does enhance the surface delivery of AMPARs, although its expression in stargazer granule cells rescues the surface expression levels of AMPARs only to a small extent [14,51,68,70]. The knockout of γ7 leads to a decreased number of all AMPARs in cerebellar extracts, with the biggest reduction found in Bergmann glia cells [71]. Surprisingly, the knockdown of γ7 in stargazer cerebellar granule cells increases the synaptic AMPAR density [14,70]. This shows an inhibitory function for γ7 in the synaptic delivery of AMPARs in granule cells [14,70]. Notably, γ7 distinguishes between AMPAR subunits as well as their editing variants: It enhances the surface expression of calcium-permeable AMPARs, whereas it reduces that of calcium-impermeable AMPARs [14]. This editing-dependent regulation of membrane trafficking was also described for γ2. The calcium-impermeable receptors do not need γ2 for the plasma membrane delivery whereas calcium-permeable receptors need to be accompanied by γ2 [72]. This differential trafficking of calcium-permeable and calcium-impermeable AMPARs had already been described well before the discovery of auxiliary subunits and has recently been reviewed [73]. The calcium permeability is determined by the amino acid at the narrow constriction of the ion pore-forming loop in the subunit GluA2 (see Figure 2). In the genome-encoded, unedited version, this amino acid is a glutamine, which can be altered via RNA editing to an arginine. With the arginine in place, the entire receptor complex is rendered virtually calcium-impermeable [74]. The editing state is checked in the ER, and therefore constitutes an important ER export factor by itself.

### 3.2. Impact of Other Auxiliary Subunits on Trafficking to the Plasma Membrane

The cornichon homologues are involved in the trafficking and surface delivery of AMPARs as well. However, their influence varies greatly between different cell types, expression systems, and AMPAR subunits [34]. In HEK cells, CNIH-2 coexpression enhances the surface delivery of GluA1 up to a factor of ten, and CNIH-2 is detectable in a complex with GluA1 on the surface [29,31,45,61]. In CA1 neurons, CNIH-2 is mandatory for the surface expression of GluA1 [32], and in stargazer granule cells, it can partially rescue extrasynaptic AMPAR expression but not synaptic receptor function [31]. In hippocampal neurons, however, the overexpression of CNIH-2 has virtually no impact on AMPAR surface expression, and CNIH-2 itself is not detectable in the plasma membrane [31].

The novel auxiliary subunit GSG1L, a structural relative of TARPs and claudins, increases the surface expression level of GluA2 in HEK cells by a factor of two, an effect comparable to that of γ2 in this specific experimental setup [45]. However, further studies in heterologous expression systems and *in vivo* are required to corroborate these findings.

## 4. Synaptic Targeting and Anchoring of AMPARs

### 4.1. TARPs and Synaptic Targeting and Anchoring

Besides regulating trafficking to the plasma membrane in general, auxiliary subunits are also involved in the synaptic delivery of initially extrasynaptically located AMPARs. Type I TARPs, for instance, mediate synaptic anchoring and clustering of AMPARs [51,66,75], as demonstrated by the ability of any type I TARP to rescue the lack of not only of extrasynaptic but also synaptic AMPARs in stargazer granule cells. [13,51,76,77]. Type II TARPs, on the other hand, have more varied influences on synaptic targeting. γ5 alters neither trafficking to the plasma membrane nor synaptic localization of AMPARs in stargazer granule cells, its role seems to be merely functional modulation, whereas γ7 was initially reported to rescue only extrasynaptic but not synaptic AMPARs [31,51,69]. However, more recent data indicate that γ7 in cerebellar granule cells does in fact act synergistically with γ2 in stimulating not only the surface delivery but also the synaptic targeting of AMPARs depending on their subunit composition: γ7 seems to reduce the amount of calcium-impermeable and promote that of calcium-permeable AMPARs in extrasynaptic as well as synaptic membranes [14]. Another study of various cerebellar synapses also reported an influence of γ7 on AMPAR abundance in the membrane: Knockdown of γ7 reduced the content of most AMPAR subunits in membranes, with the reduction being more pronounced at the synapse than in extrasynaptic membranes [71].

The synaptic anchoring of AMPARs is mediated via MAGUKs such as the PSD proteins PSD-95, PSD-93, and SAP-102. These scaffolding proteins are major components of the PSD and have long been known to interact indirectly with AMPARs [15,78]. The overexpression of PSD-95 in particular leads to enhanced AMPAR but not NMDAR synaptic responses in hippocampal or cortical neurons caused by a selective increase in the number and the clustering of synaptic AMPARs [66,79,80,81]. Thus, PSD-95 controls the AMPAR content at the synapse, which provides the basis for LTP generation in corticocortical synapses [66,79,81,82]. The knockdown of PSD-95 or PSD-93 silences large, non-overlapping populations of synapses, predominantly in the mature brain, whereas SAP-102 is more important for the synaptic clustering of AMPARs in the immature brain [83]. However, within mature PSD-95/-93 double knockout mice, SAP-102 is upregulated to compensate for the loss [83].

AMPARs cannot interact directly with PSD-95 because they lack a PDZ binding motif that is required for the binding to the PDZ domains of synaptic scaffolding proteins such as PSD-95 [15,84]. Type I TARPs contain such a PDZ binding motif (TTPV) at their very C-terminus that binds with the same affinity to all three PDZ domains of PSD-95 [85]. Thus, TARPs are the mediators between AMPARs and PSD-95 [75,84]. AMPARs and TARPs form a stable complex that diffuses in and out of the PSD [9,86]. Once this AMPAR-TARP complex hits a PDZ domain-containing synaptic scaffolding protein, the TARP’s PDZ binding motif binds to this protein and anchors the whole complex in the PSD. There might be a competition between various synaptic scaffolding proteins such as PSD-95/-93, MAGI-2, and other MAGUK proteins for binding to the AMPAR-TARP complex, which may serve as some sort of control mechanism, but these protein-protein interactions still need further investigation [83,87].

The efficacy of γ2 binding to PSD-95 is highly dependent on the phosphorylation of the TARP’s C-terminal domain, which contains one threonine and nine serine phosphorylation sites [45,88,89]. Phosphorylation of the threonine (321) located within the PDZ binding motif of γ2 completely abolishes the interaction between γ2 and PSD-95 [88,89]. For the serine phosphorylation sites, the opposite holds true: They need to be phosphorylated to enable binding of γ2 to PSD-95. This effect can be explained by the binding of the non-phosphorylated TARP C-terminus to the negatively charged phospholipids of the plasma membrane, which renders it inaccessible to the PDZ domain of PSD-95 [90,91]. Upon serine phosphorylation, the interaction of the TARP C-terminus with the membrane is abolished, and the PDZ binding motif becomes accessible [91]. Consequently, a mutant γ2 lacking all C-terminal serine phosphorylation sites still enhances the plasma membrane expression of AMPA receptors but is not capable of anchoring them in the PSD [92]. Likewise, γ2 with dephosphorylated C-terminal serines shows diffuse localization in the plasma membrane, whereas phosphorylated γ2 is located at the PSD [90,92,93]. These phosphorylation mechanisms play an important role in the regulation of LTP and LTD [92]. For example, the dephosphorylation of the C-terminal serines of TARPs is a common LTD pathway, and at mutant γ2 lacking all C-terminal serine phosphorylation sites prevents cerebellar and hippocampal LTP [67,92,94]. 

The serines of the C-terminus of γ2 are phosphorylated by CaMKII as well as PKC and are dephosphorylated by PP1 and PP2b [92]. A correlation between PKC activity and AMPAR activity had been discovered well before TARPs were recognized as auxiliary subunits [95]. CaMKII translocates to the PSD upon activation of NMDA-type glutamate receptors, phosphorylates γ2, and thus triggers diffusional trapping of AMPARs at the synapse, which means a translocation of the receptor to CaMKII-free synapses or extrasynaptic sites [96,97]. The phosphorylation at threonine 321 within the PDZ binding motif by PKA and MAPKs has the opposite effect, decreasing AMPAR clustering and anchoring at the synapse [98]. These phosphorylation and the respective dephosphorylation mechanisms as well as the proteins involved are partly different for different TARPs. For example, γ8 is dephosphorylated by PP1 and PP2A, γ2 by PP1 and PP2B [86]. The type II TARP γ5 lacks most of the phosphorylation sites as well as the classical PDZ binding motif, which are conserved only within type I TARPs. Thus, γ5 cannot bind to PSD-95 and therefore does not anchor AMPARs at the PSD [99].

The C-terminus of γ2 potentially plays a role during the entire trafficking process of the receptor because it interacts with nPIST, which is a Golgi-resident PDZ protein that cycles with the TARP-AMPAR complex between the Golgi apparatus, the plasma membrane, and the PSD [100].

**Table 1:** Modulation of AMPARs by TARPs; +, a positive modulation or interaction; −, a negative modulation; (+)/(−), possible or expected positive or negative modulation; 0, no modulation; ~, unclear/different results described; N/A, no data available. Contradictory results and observations are discussed in the text.

Level of Influence	Interaction with AMPARs	Agonist Efficacy	Amplitude		Desensitization/Activation	ER/Golgi Export/Traffick	Maturation		Plasma Membrane Expression	Synaptic Targeting/Anchoring
γ1	N/A	0 ^A^	0 ^B^		N/A	N/A	N/A		0 ^C^	N/A
γ2	+ ^D^	+ ^E^	+ ^F^		− ^G^	+ ^H^	+ ^I^		+ ^J^	+ ^K^
γ3	+ ^L^	+ ^M^	+ ^N^		− ^O^	(+) N/A ^P^	+ ^Q^		+ ^R^	(+) N/A ^S^
γ4	+ ^T^	+ ^U^	+ ^V^		− ^W^	(+) N/A ^X^	(+) N/A ^Y^		+ ^Z^	(+) N/A ^AA^
γ5	+ ^AB^	− ^AC^	+ ^AD^		+ ^AE^	N/A	N/A		~ ^AF^	~ ^AG^
γ6	N/A	N/A	0 ^AH^		N/A	N/A	N/A		N/A	N/A
γ7	+ ^AI^	+ ^AJ^	+ ^AK^		− ^AL^	N/A	N/A		+ ^AM^	+ ^AN^
γ8	+ ^AO^	+ ^AP^	+ ^AQ^		− ^AR^	(+) N/A ^AS^	(+) N/A ^AT^		+ ^AU^	+ ^AV^
A: [21]				Q: [54]				AG: [13,67,69,99]		
B: [13,21]				R: [50,51,54]				AH: [68,69]		
C: [21,50]				S: [9,71,75,88,94,104]				AI: [18,34,68,69,99]		
D: [9,10,13,55,75,88]				T: [13]				AJ: [62,68]		
E: [10,17,19,20,21,22,51,62,67,68,101]				U: [10,17,21,22,51,67,101,102]				AK: [34,62,69]		
F: [10,13,18,19,20,21,22,34,51,62,69,92,101,102,103]				V: [10,13,20,21,22,34,51,101,102,103]				AL: [14,68]		
G: [6,10,18,19,21,51,55,67,68,101,102]				W: [10,21,51,101,102]				AM: [14,51,68,71]		
H: [13,16,50,55]				X: [13,16,50,55]				AN: [14,51,68,71]		
I: [13,16,45,50,54]				Y: [13,16,45,50,54]				AO: [13]		
J: [9,19,21,23,50,54,55,67,71,88,104,105]				Z: [51]				AP: [10,17,20,21,22,34,62,67,102]		
K: [9,71,75,88,94,104]				AA: [9,71,75,88,94,104]				AQ: [10,13,21,22,34,51,62,65,102,103]		
L: [7,13]				AB: [13,18,34,68,69,99]				AR: [10,34,51,62,102]		
M: [10,17,21,22,51,67,102]				AC: [7,67,69]				AS: [13,16,50,55]		
N: [10,13,20,21,22,34,51,102,103]				AD: [13,34,68,69,99]				AT: [13,16,45,50,54]		
O: [10,21,51,102]				AE: [68,69,99]				AU: [34,65,71,106]		
P: [13,16,50,55]				AF: [13,67,69,99]				AV: [34,65,71,106]		

**Table 2:** Modulation of AMPARs by auxiliary subunits other than TARPs; +, a positive modulation or interaction; −, a negative modulation; (+)/(−), possible or expected positive or negative modulation; 0, no modulation; ~, unclear/different results described; N/A, no data available. Contradictory results and observations are discussed in the text. (des. = desensitization; deac. = deactivation).

Level of Influence	Interaction with AMPARs	Agonist Efficacy		Amplitude	Desensitization/Activation		ER/Golgi Export/Traffick.	Maturation		Plasma Membrane Expression	Synaptic Targeting/Anchoring
Stg-1	+ ^A^	N/A		+ ^B^	− ^C^		N/A	N/A		0^D^	N/A
SOL-1	+ ^E^	N/A		+ ^F^	− ^G^		N/A	N/A		0^H^	N/A
SOL-2	+ ^I^	+ ^J^		+ ^K^	+ ^L^		N/A	N/A		0^M^	N/A
CNIH-2	+ ^N^	~ °		+ ^P^	~ ^Q^		+ ^R^	+ ^S^		0 ^T^	~ ^U^
CNIH-3	+ ^V^	0 ^W^		+ ^X^	− ^Y^		+ ^Z^	+ ^AA^		0 ^AB^	~ ^AC^
CKAMP44	+ ^AD^	+ ^AE^		N/A	+ des. ^AF^ − deac.		N/A	N/A		0 ^AG^	(+) N/A ^AH^
SynDIG–1	+ ^AI^	0 ^AJ^		0 ^AK^	0 ^AL^		N/A	N/A		N/A	+ ^AM^
GSG1L	+ ^AN^	N/A		+ ^AO^	− ^AP^		N/A	N/A		+ ^AQ^	N/A
A: [24]			L: [28]			W: [35]			AH: [42,44]		
B: [24,25]			M: [28]			X: [29,35]			AI: [40,108]		
C: [25]			N: [29,32,34,107]			Y: [6,29,35]			AJ: [41]		
D: [24,25]			O: [31,34,35,62,102]			Z: [30,58,60,61,62]			AK: [41]		
E: [25,26,27,28]			P: [29,31,34,35,61,62,102]			AA: [32]			AL: [41]		
F: [25,26,27,28]			Q: [6,31,34,35,62,102]			AB: [29]			AM: [39,40,108]		
G: [25]			R: [30,58,60,61,62]			AC: [29,31,34]			AN: [6,45]		
H: [24,25,26,27]			S: [32]			AD: [42]			AO: [6]		
I: [28]			T: [29,31,34,61]			AE: [42]			AP: [6,45]		
J: [28]			U: [6,29,31]			AF: [42,43]			AQ: [45]		
K: [28]			V: [29]			AG: [42,43]					

The deletion of the C-terminus including the PDZ-binding motif of γ2 abolishes all synaptic trafficking but not the plasma membrane delivery for most AMPARs [75]. In keeping with this result, overexpression of γ2 lacking the C-terminal domain (γ2-∆C) or at least the terminal PDZ binding motif in stargazer hippocampal neurons increases the number of extrasynaptic AMPARs [75,92]. In non-stargazer hippocampal neurons, AMPAR activity is reduced upon overexpression of γ2-∆C, and the surface mobility of the receptor-TARP complex is increased [9]. The PDZ-binding motif is also important for γ8 function because the overexpression of γ8 without the PDZ-binding motif in hippocampal pyramidal cells decreases AMPAR activity [109]. 

There are additional regulatory mechanisms that impact the binding of TARPs to AMPARs and PSD-95. The C-terminus of γ2 but not γ8 can be truncated by the protease calpain. Therefore, the binding of γ2 to PSD-95 can be permanently interrupted by increased calpain activity [110]. Furthermore, a cysteine residue at position 302 within the C-terminal domain of γ2 can be nitrosylated, which augments the TARP-AMPAR interaction and ultimately leads to increased surface delivery of the receptor [111]. Palmitoylation of PSD-95 appears to be required for AMPAR anchoring at the PSD, as demonstrated by a selective loss of synaptic AMPARs following inhibition of PSD-95 palmitoylation in hippocampal neurons [112]. As suggested by heterologous expression studies in COS cells, this loss is caused by a disruption of PSD-95 clustering with TARPs and AMPARs.

### 4.2. The Impact of Other Auxiliary Subunits on Synaptic Targeting and Anchoring

There is no evidence that CNIH-2 influences synaptic targeting; apparently, it is only involved in extrasynaptic trafficking of AMPARs [31]. SynDIG1, on the other hand, promotes the trafficking of AMPARs to synapses and is involved in their synaptic clustering [39,108]. SynDIG1 cycles between the extrasynaptic plasma membrane, excitatory synapses, and intracellular endosomal compartments. Given its direct interaction with AMPARs, there is a good chance that SynDIG1 is accompanied by AMPARs during cycling [39,40]. 

CKAMP44 is most likely involved in the synaptic anchoring of AMPARs and it occurs probably the same way as for TARPs, because CKAMP44, just like TARPs, can interact with PSD-95 [44].

## 5. Conclusions and Outlook

For the trafficking of AMPARs to their synaptic sites of action, the presence of auxiliary subunits like TARPs or CNIHs is mandatory. TARPs and CNIHs directly interact with the AMPARs, starting immediately after translation in the ER or at least after early maturation, *i.e.*, final modification of their glycosylation and receptor assembly. They influence receptor subunit composition and promote the export of receptor complexes from the ER and Golgi apparatus. TARPs are closely associated with AMPARs in the plasma membrane where they function as mediators between the receptor and scaffolding proteins such as PSD-95. The interaction between scaffolding proteins and TARPs is mainly regulated by the phosphorylation state of the TARP’s C-terminal domain and determines the anchoring and location of the receptor at the PSD.

While the impact of TARPs on the trafficking of AMPARs and the regulation of this process are relatively well known through a considerable number of recently published thorough studies, the modulatory influence of the other AMPAR auxiliary subunits identified to date like the CNIHs, the SynDIG protein family, the shisa protein family, and GSG1L is not yet well understood. Even though it is clear that CNIHs and GSG1L enhance the trafficking of AMPARs, the extent of their impact, the regulatory mechanisms, and the interaction sites are all still unknown. In addition, it is rather unlikely that the full complement of AMPAR auxiliary subunits has already been identified. Thus, additional proteins can be expected to come into play. Possible future players in this still growing field of AMPAR modulators include certain members of the claudin family, which are suspected to be “type III TARPs” with their own specialized modulatory effects on AMPARs [113].

An entirely open field, of which we have barely scratched the surface, is the question whether and how these multiple auxiliary subunits interact or compete with each other for AMPA receptor modulation. These questions urgently need to be addressed in future studies of these important proteins.

## Definitions

AMPA: α-amino-3-hydroxy-5-methyl-4-isoxazole propionic acid
AMPAR: AMPA receptor
AP-4: adaptor protein-4
CAMKII: Ca^2+^/calmodulin-dependent protein kinase II
CKAMP: cysteine-knot AMPAR modulating protein
CNI: cornichon
CNIH: cornichon homologue
CNS: central nervous system
ER: endoplasmic reticulum
γ2-ΔC: γ2 lacking the C-terminal domain
GLR: glutamate receptor-like protein
GSG1L: germ cell-specific gene 1-like
HEK: human embryonic kidney
iGluR: ionotropic glutamate receptor
LC2: light chain protein 2
LTD: long-term depression
LTP: long-term potentiation
MAGI-2: membrane-associated guanylate kinase inverted-2
MAGUK: membrane-associated guanylate kinase
MAP1A: microtubule-associated protein 1A
MAPK: mitogen-activated protein kinase
mEPSP: miniature excitatory postsynaptic potential
Neto: neuropilin and tolloid-like
NMDA: *N*-methyl-d-aspartate
NMDAR: NMDA receptor
nPIST: neuronal isoform of protein interacting specifically with TC10
PKC: protein kinase C
PDZ: PSD-95/discs large/zonula occludens-1
PP: protein phosphatase
PSD: postsynaptic density
SAP: synapse-associated protein
SOL: suppressor of lurcher
SynDIG: synapse differentiation-induced gene I
TARP: transmembrane AMPA receptor regulatory protein
